# Sources of performance variability in deep learning-based polyp detection

**DOI:** 10.1007/s11548-023-02936-9

**Published:** 2023-06-02

**Authors:** T. N. Tran, T. J. Adler, A. Yamlahi, E. Christodoulou, P. Godau, A. Reinke, M. D. Tizabi, P. Sauer, T. Persicke, J. G. Albert, L. Maier-Hein

**Affiliations:** 1 Division of Intelligent Medical Systems, DKFZ, Heidelberg, Germany; 2 Faculty of Mathematics and Computer Science, University of Heidelberg, Heidelberg, Germany; 3 Interdisciplinary Endoscopy Center (IEZ), University Hospital Heidelberg, Heidelberg, Germany; 4 Department of Gastroenterology, Hepatology and Endocrinology, Robert-Bosch Hospital (RBK), Stuttgart, Germany; 5 Clinic for General Internal Medicine, Gastroenterology, Hepatology and Infectiology, Pneumology, Klinikum Stuttgart, Stuttgart, Germany; 6 National Center for Tumor Diseases (NCT), NCT Heidelberg, a Partnership Between DKFZ and University Medical Center Heidelberg, Heidelberg, Germany

**Keywords:** Validation, Evaluation, Metrics, Object detection, Surgical data science, Variability

## Abstract

**Purpose:**

Validation metrics are a key prerequisite for the reliable tracking of scientific progress and for deciding on the potential clinical translation of methods. While recent initiatives aim to develop comprehensive theoretical frameworks for understanding metric-related pitfalls in image analysis problems, there is a lack of experimental evidence on the concrete effects of common and rare pitfalls on specific applications. We address this gap in the literature in the context of colon cancer screening.

**Methods:**

Our contribution is twofold. Firstly, we present the winning solution of the Endoscopy Computer Vision Challenge on colon cancer detection, conducted in conjunction with the IEEE International Symposium on Biomedical Imaging 2022. Secondly, we demonstrate the sensitivity of commonly used metrics to a range of hyperparameters as well as the consequences of poor metric choices.

**Results:**

Based on comprehensive validation studies performed with patient data from six clinical centers, we found all commonly applied object detection metrics to be subject to high inter-center variability. Furthermore, our results clearly demonstrate that the adaptation of standard hyperparameters used in the computer vision community does not generally lead to the clinically most plausible results. Finally, we present localization criteria that correspond well to clinical relevance.

**Conclusion:**

We conclude from our study that (1) performance results in polyp detection are highly sensitive to various design choices, (2) common metric configurations do not reflect the clinical need and rely on suboptimal hyperparameters and (3) comparison of performance across datasets can be largely misleading. Our work could be a first step towards reconsidering common validation strategies in deep learning-based colonoscopy and beyond.

## Introduction

Colorectal cancer is one of the most common cancer types, ranking second in females and third in males [1]. By detecting and subsequently resecting neoplastic polyps during screening colonoscopy, the risk of developing the disease can be reduced significantly. Research focuses on developing deep learning (DL) solutions for automated detection of polyps in colonoscopy videos [2–6]. However, to date, the metrics with which algorithms are validated receive far too little attention. These metrics are not only important for measuring scientific progress, but also for gauging a method’s potential for clinical translation. While previous work has highlighted general metric pitfalls in the broader context of classification, segmentation and detection [7], we are not aware of any prior studies systematically analyzing common metrics in the context of polyp detection. Our underlying hypothesis was that reported performance values in polyp detection methods are largely misleading as they are sensitive to many validation design choices including (1) the choice of test set and (2) the chosen metric configurations (e.g., threshold for the localization criteria). Our contribution is twofold: Firstly, we present the winning solution of the Endoscopy Computer Vision Challenge (EndoCV) on colon cancer detection, conducted in conjunction with the IEEE International Symposium on Biomedical Imaging (ISBI) 2022. Secondly, based on publicly available challenge data, we demonstrate the sensitivity of commonly used metrics to a range of hyperparameters as well as the consequences of poor metric choices.

## Methods

Here, we present the winning method of the EndoCV challenge on colon cancer detection, conducted in conjunction with ISBI 2022 (Sect. 2.1), and revisit common detection metrics including their hyperparameters (Sect. 2.2).

### Object detection algorithm

We base our study on a state-of-the-art detection method, namely the winning entry [8] of the EndoCV 2022 polyp detection challenge [4].

#### Method overview

**Fig. 1:**
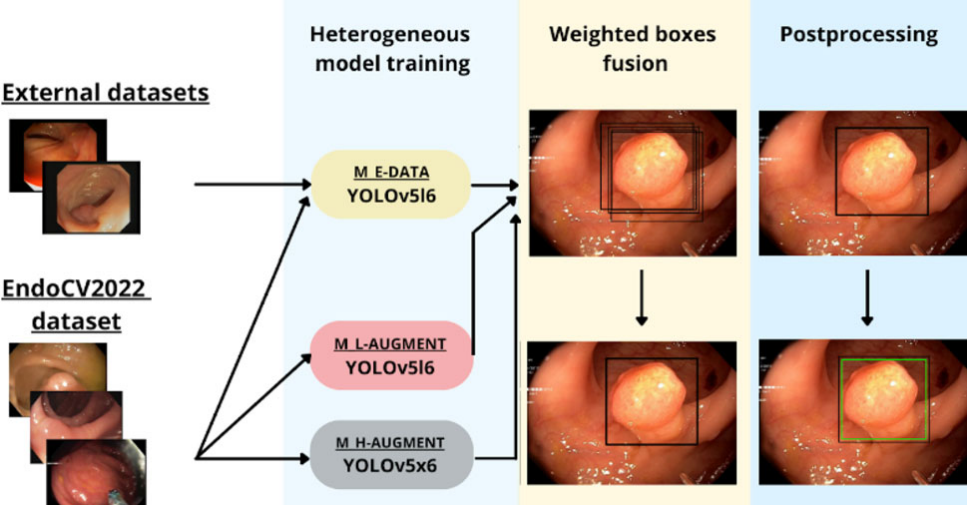
Winning submission of the Endoscopy computer vision challenge (EndoCV) on colon cancer detection. We used a YOLOv5 backbone and specifically chose the large (YOLOv5l6) and extra-large (YOLOv5x6) version. For three specific settings, namely light augmentation (M L-AUGMENT), heavy augmentation (M H-AUGMENT), and inclusion of external data (M E-DATA), we ensembled two models trained on two different folds each. The ensemble predicts a set of bounding box candidates, which were merged using weighted boxes fusion and postprocessed to yield the final prediction

The method is illustrated in Fig. 1. A heterogeneous ensemble of YOLOv5-based models was trained on the EndoCV2022 challenge training dataset [4], a collection of 46 endoscopic video sequences of polyps. The median length of each video was 51 frames with 3290 frames in total, of which 2631 contained polyps. For training, we split the data into disjoint subsets. Originally, we created four subsets for stratified four-fold cross-validation, but the final ensemble consisted of models which were trained on only two of the four folds to decrease training and inference time. Furthermore, on each fold we trained three models for the ensemble with complementary training setups, as follows: the model was trained with (1) only light data augmentation (horizontal flips, scale, color jitter, and mosaic), (2) heavy data augmentation (light augmentations, vertical flips, translation, rotation, mix-up, shear, and copy-paste augmentations), and (3) light data augmentation, but additional training data from external polyp datasets (CVC-Datasets, Etis-Larib) [9–11] were added (see [8] for details). Overall, this led to six ensemble members (two folds, three models each). As test sets, we used appropriate subsets of the PolypGen dataset [12] to highlight the variability of the metrics. The dataset consists of a database of single-frame endoscopic images of polyps from six data centers, each with a median of 242 frames, totaling 1512 single testing frames. Details about the exact number of frames and polyp prevalence for each center can be found in Sect. 3.1, Table 2.

#### Implementation details

The models were trained for 20 epochs using a stochastic gradient descent optimizer, a learning rate of 0.1, and a *complete intersection over union* [13] loss. The non-maximum suppression algorithm was applied to each ensemble member individually with an *intersection over union (IoU)* threshold of 0.5. The individual member predictions were merged using the weighted boxes fusion [14] (WBF) algorithm. For the WBF hyperparameters, we chose an *IoU* threshold of 0.5, a skip box threshold of 0.02, and all models were weighted equally. As we observed a tendency towards oversegmentation, we shrank all bounding boxes with a confidence score higher than 0.4 by 2% of their size during post-processing.

### Object detection metrics

Three metric-related design decisions are important when assessing performance of object detection algorithms [15]:

1. *Localization criterion* The localization criterion determines whether a predicted object spatially corresponds to one of the reference objects and vice versa by measuring the spatial similarity between prediction (represented by a bounding box, pixel mask, center point or similar) and reference object. It defines whether the prediction hit/detected (true positive) or missed (false positive) the reference. Any reference object not detected by the algorithm is defined as false negative. The localization criteria that were applied in this work comprise two groups, namely the point-based criteria and the overlap-based criteria (Appendix A).
2. *Assignment strategy* As applying the localization criterion might lead to ambiguous matchings, such as two predictions being assigned to the same reference object, an assignment strategy needs to be chosen that determines how potential ambiguities are resolved. As multiple polyps in the same image are rather rare, an assignment strategy is not as relevant as in other applications. With respect to the metric configuration, we therefore focus on the localization criterion and the classification metrics.
3. *Classification metric* Based on the choice of localization criterion and assignment strategy, standard classification metrics can be computed at object level [7]. The most popular multi-threshold metric in object detection is *Average Precision (AP)* (Fig. 9).

As a foundation of this work, we determined common metrics in object detection challenges, along with their respective localization criterion and classification metric (Table 1).

**Table 1:** Common design choices for validation of polyp detection methods according to international competitions

	Localization criterion	Classification metric
EndoVis 2015 [16]	*Point inside mask*	*PPV*
		*Sensitivity*
		*Overall/Average F1-score*
GIANA 2017 [6]	*Point inside mask*	*PPV*
GIANA 2018 [6]		*Sensitivity*
		*F1/F2-score*
		Custom metrics
GIANA 2021 [5]	*Box IoU* (only 2021)	*AP@0.5:0.95* (only 2021)
EndoCV 2021 [3]	*Box IoU*	*AP@0.5, AP@0.75, AP@0.5:0.95*
EndoCV 2022 [4]		*AP* *across 3 scales of polyp size*
		*Mean of 4* ***AP***s

*PPV* positive predictive value, *IoU* intersection over union, *AP* average precision

## Experiments and results

In this section, we investigate the sensitivity of popular classification metrics to the test set composition (Sect. 3.1) and the localization criterion (Sect. 3.2). We further assess the clinical value of commonly used metric configurations (Sect. 3.3). Details on the metrics and localization criteria are provided in Appendix A.

### Effect of test set

In the following, we quantitatively assess the performance variability resulting from the chosen test set, specifically from the target domain (i.e., the clinical validation center) and the distribution of polyp size.

#### Sensitivity to center

**Fig. 2:**
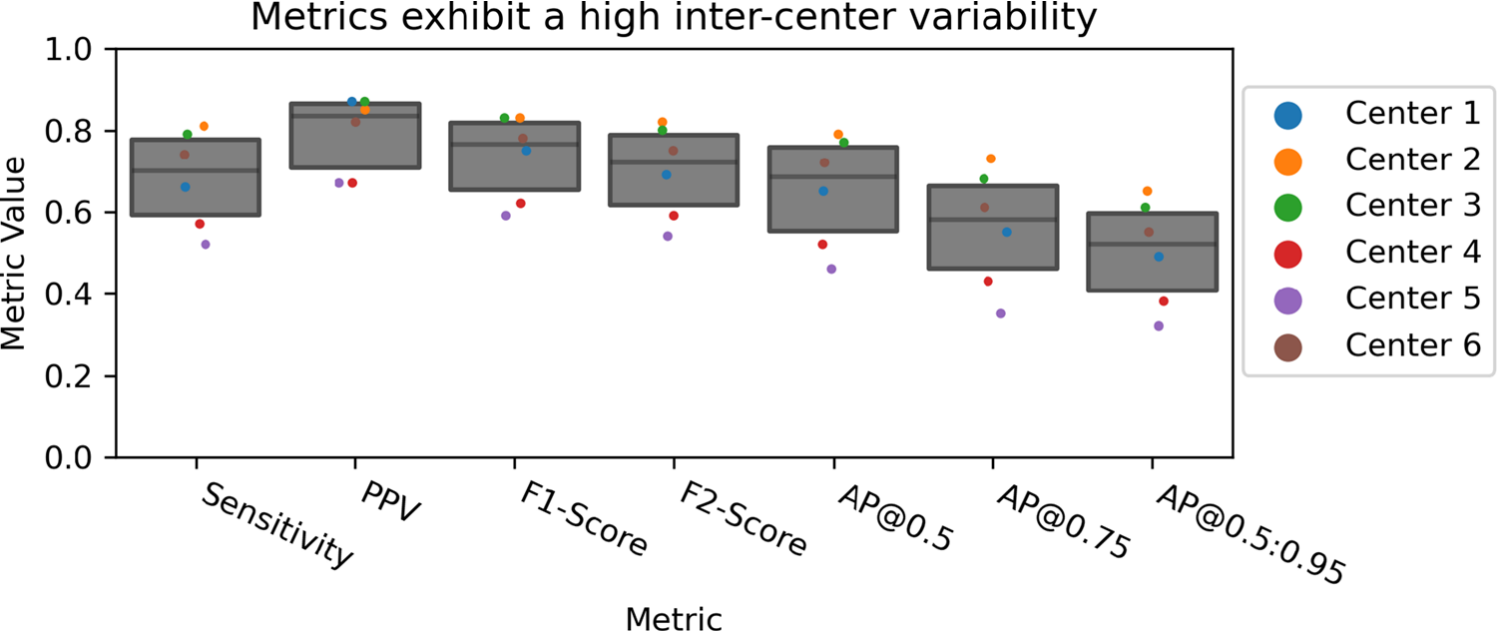
Performance variability resulting from the chosen validation center. *Sensitivity*, *PPV*, *F1-Score* and *F2-Score* are calculated using a *Box IoU* as criterion and a cutoff threshold of 0.5. The *AP* is calculated for thresholds 0.5, 0.75 and threshold range 0.5:0.95. All commonly used classification metrics (cf. Table 1) show a substantial sensitivity to the center. The dot-and-box plots contain aggregated values per center. *PPV* positive predictive value, *AP* average precision, *IoU* intersection over union

To show the variability of performance resulting from different test sets, we used data from six validation centers [12]. Figure 2 shows the performance of our object detection method (Sect. 2.1) according to commonly used metrics, using Box IoU as criterion and cutoff threshold 0.5. These exhibit high variability between centers. For example, the *AP*@0.5:0.95 ranges from [0.38, 0.63], which is notable, given that the *AP* of the top three submissions for EndoCV 2022 ranged from [0.12, 0.33].

#### Sensitivity to polyp size

We further calculated the *AP* scores from all six validation centers, stratified by polyp size (Table 2). The polyp sizes are defined in pixels for images with $1920 \times 1080$ pixel resolution. Polyps smaller than $100^2$ pixels are considered small, polyps larger than $200^2$ pixels are counted as large and the rest are counted as medium sized, as suggested in the EndoCV2021 challenge [3]. A high variability can be observed, indicating that algorithm performance is highly affected by the distribution of polyp sizes.

**Table 2:** *AP* stratified by polyp size. The results are shown for a fixed *IoU* threshold of 0.5 (left) as well as for a range of thresholds following the COCO benchmark evaluation standard [17, 18] (center)

Metric	*AP@0.5*			*AP@0.5:0.95*			*n*	$\phi (\%)$
Polyp size	Small	Medium	Large	Small	Medium	Large		
Center 1	0.24	0.48	0.73	0.14	0.33	0.55	256	98
Center 2	0.00	0.46	0.81	0.00	0.31	0.67	276	89
Center 3	0.16	0.64	0.91	0.10	0.47	0.72	457	99
Center 4	0.14	0.33	0.59	0.06	0.27	0.43	227	64
Center 5	0.19	0.39	0.59	0.12	0.26	0.42	208	99
Center 6	0.00	0.52	0.89	0.00	0.39	0.67	88	94
All centers (SD)	0.14 (0.1)	0.47 (0.11)	0.75 (0.14)	0.07 (0.06)	0.34 (0.08)	0.58 (0.13)	1512	91

We provide additional information on the number of frames (n) and polyp prevalence ($\phi$) per center (right). *AP* average precision, *IoU* intersection over union, *SD* standard deviation

To further evaluate how the *IoU* values relate to polyp size and polyp type and simultaneously account for the hierarchical structure of the data set, we fit a linear mixed effects model (R version 4.1.3, package lme4). In this model, polyp size (small, medium, or large) and polyp type (flat or protruded) were fixed effects, while data center, patient identifier (ID), and image ID were random effects. The results suggest that there are strong effects of polyp type and polyp size on the *IoU* values. In particular when the polyp is of a protruded as opposed to a flat type, the values of *IoU* are on average higher by a difference of 0.08 (conditional that the other predictors remain constant). When the polyp is of a medium or small size compared to a large size, the *IoU* values are lower by a difference of 0.05 and 0.28, respectively (conditional that the remaining predictors remain constant).

### Effect of metric configuration

**Fig. 3:**
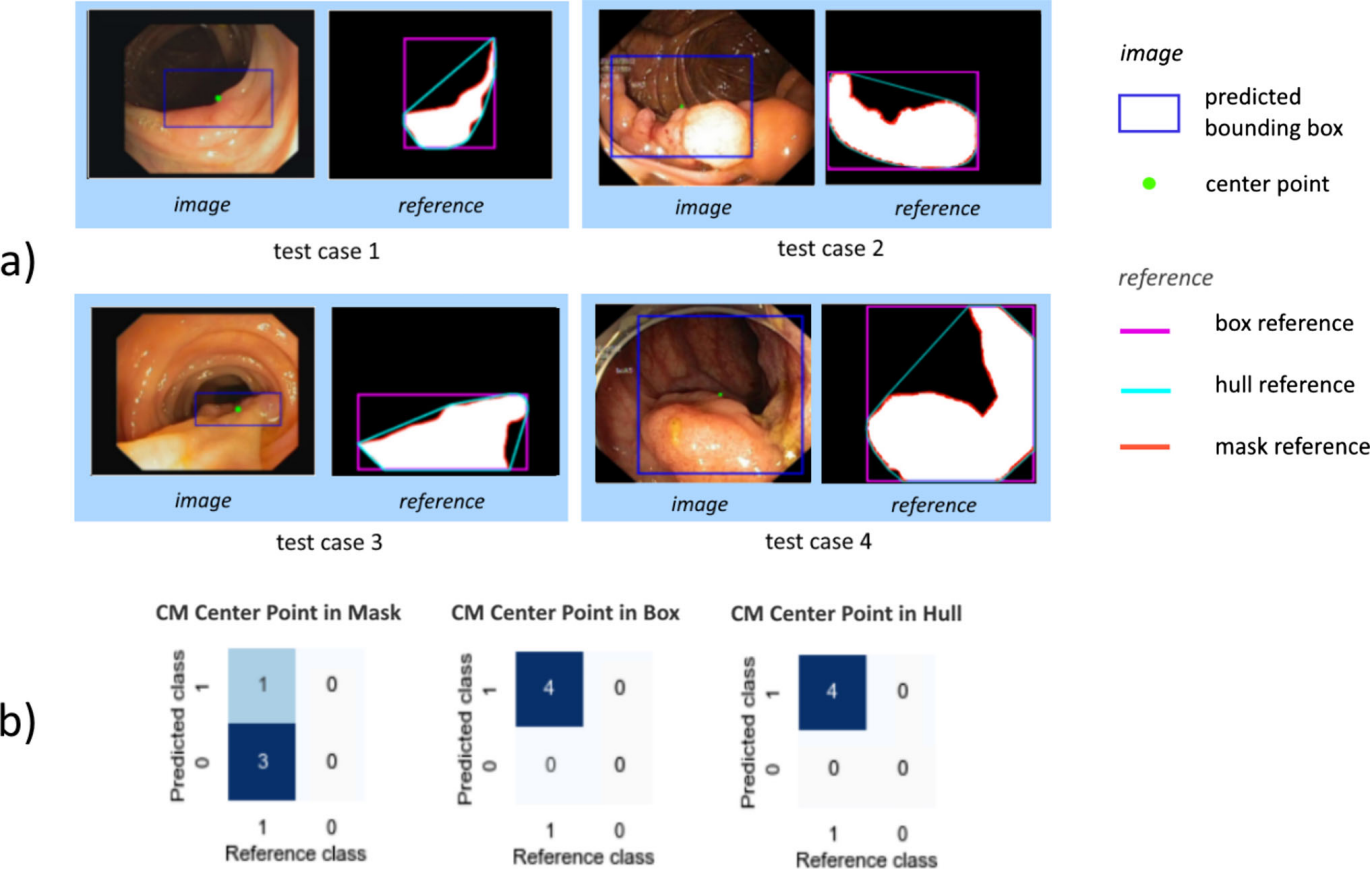
Effect of the reference shape in point-based localization criteria (**a**) on the CM (**b**). In the case of non-convex polyps, *Center Point in Mask* leads to substantially more predictions being categorized as false negatives. *CM* confusion matrix

**Fig. 4:**
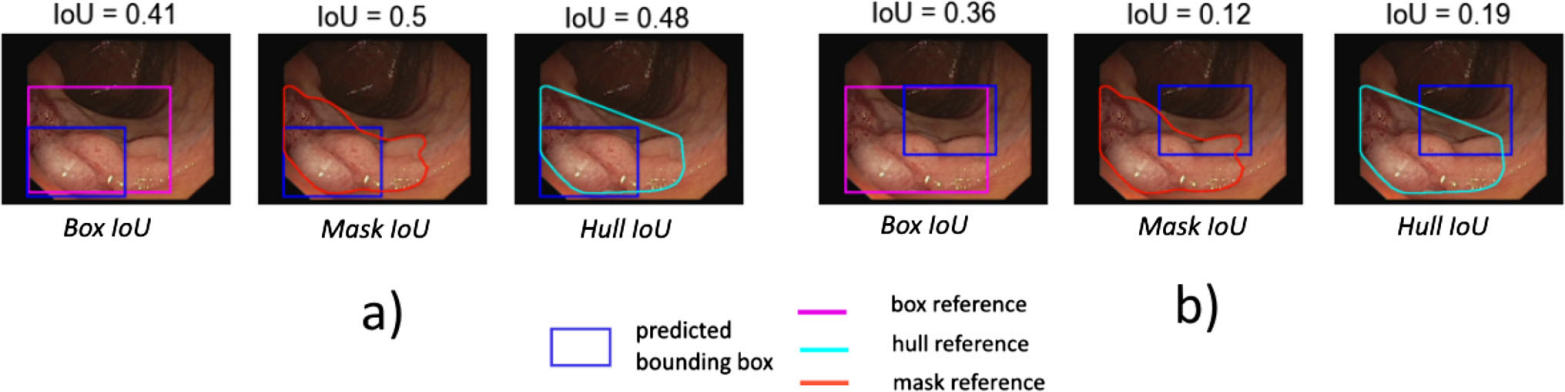
Effect of the reference shape (here: reference mask or its bounding box or convex hull) in boundary-based localization criteria. For two different (blue) predictions (**a**) and (**b**) the *IoU* results are shown. These vary substantially in the case of the inferior prediction (**b**). *IoU* intersection over union

**Fig. 5:**
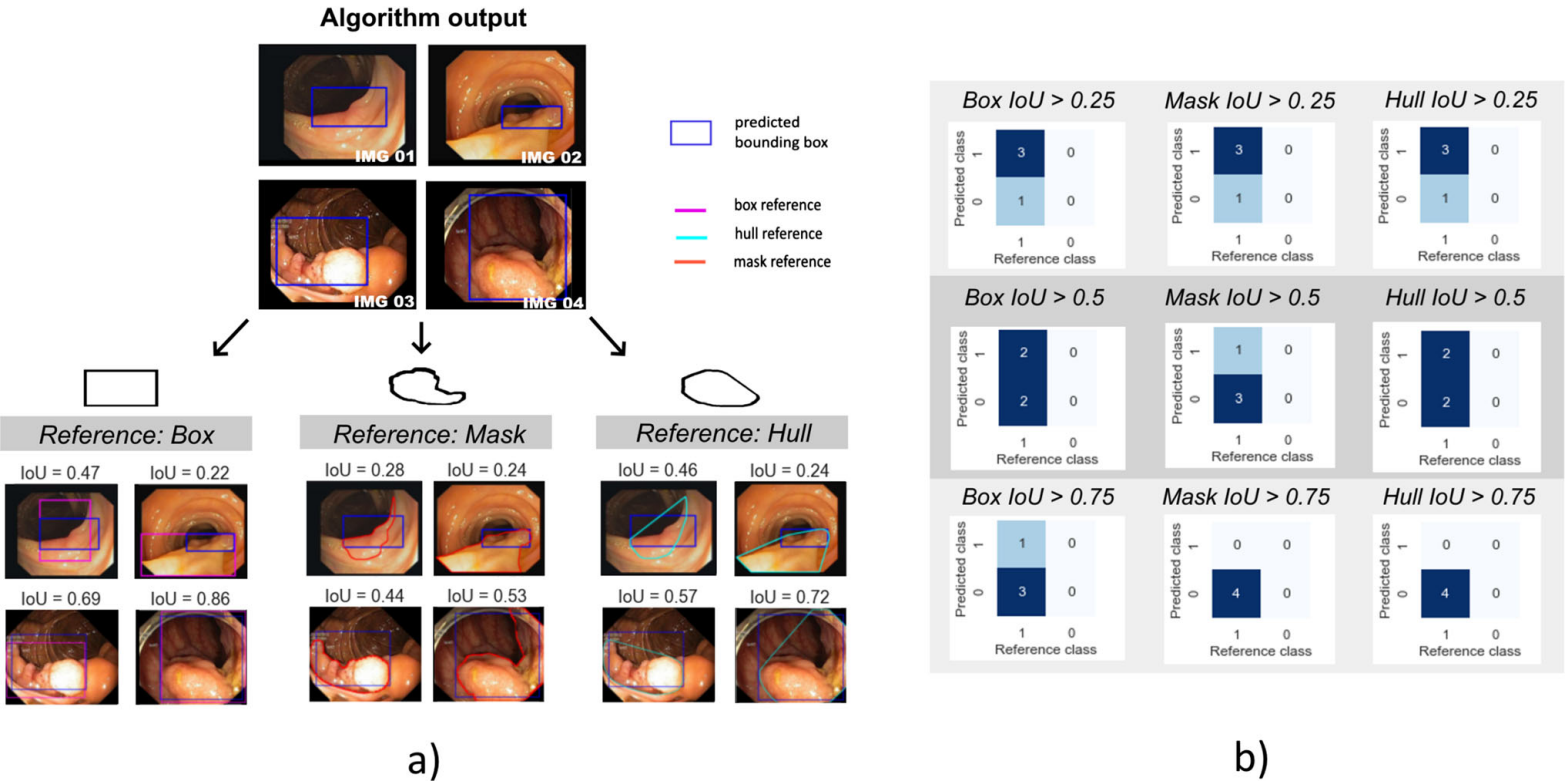
Effect of *IoU* threshold on the confusion matrix for three different overlap-based localization criteria. The same predictions produce substantially different confusion matrices for commonly used thresholds 0.5 and 0.75. *IoU* intersection over union

In the case of polyp detection, the goal of high sensitivity (not missing a polyp) is an indispensable priority. We therefore assess the effect of design choices related to the localization criterion on the decision whether a prediction is determined to be a true or false positive. Figures 3, 4 showcase the effect of the reference shape in point-based and overlap-based localization criteria, respectively, while Fig. 5 demonstrates the sensitivity of overlap-based criteria to different localization thresholds. In the following, we provide experimental evidence for the showcased phenomena.

#### Sensitivity of the AP to the specific choice of overlap-based localization criterion

**Fig. 6:**
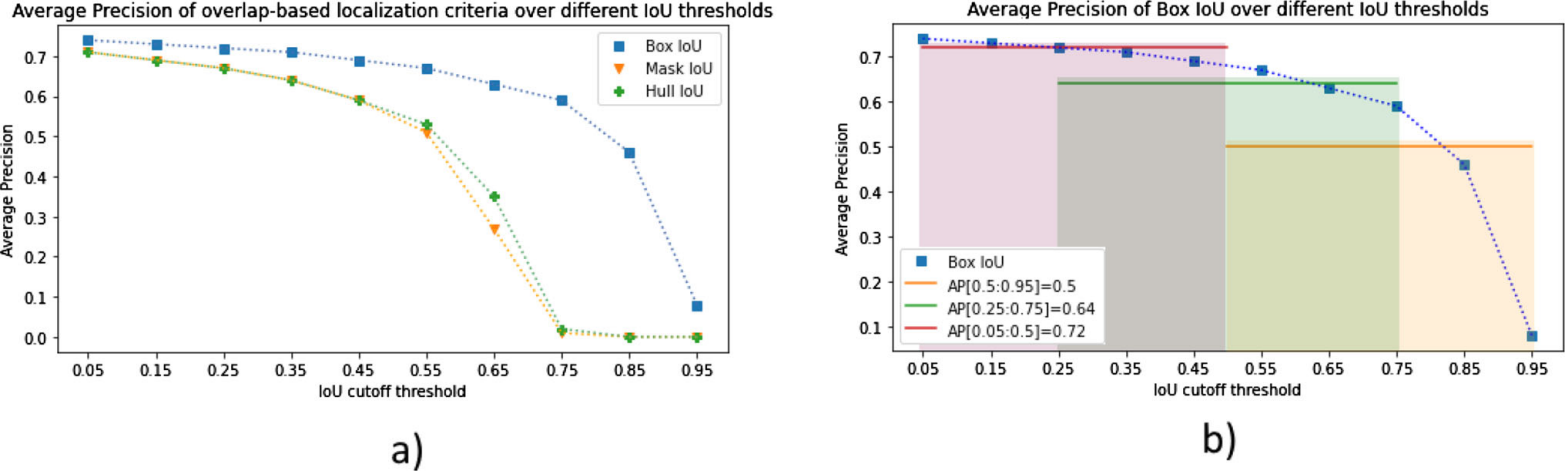
**a** Effect of different localization criteria on the most common object detection metric *AP*. Three common overlap-based criteria using different references (box, mask and hull) are plotted as a function of the *IoU* cutoff threshold in the range [0.05:0.95]. *Box IoU* scores are higher across all thresholds, while *Mask IoU* and *Hull IoU* do not differ substantially. **b** *AP* with *IoU* threshold for three different ranges of *IoU* thresholds. Note that the range [0.5:0.95] (orange) is the most common one in the computer vision community. *AP* average precision, *IoU* intersection over union

In this experiment, we investigated the *AP* scores using *Box IoU, Mask IoU* and *Hull IoU* criteria over a range of *IoU* thresholds [0.05:0.95]. The resulting curves are shown in Fig. 6a). We observe that the *Mask IoU* and *Hull IoU*-based *AP* scores are very similar; conversely, using *Box IoU* yielded overall higher *AP*, even at lower thresholds.

#### Sensitivity of the AP to the IoU range

We investigated the *AP* scores, using *Box IoU* as a criterion, over different *IoU* threshold ranges including the commonly used range of [0.5:0.95]. As shown in Fig. 6b), the *AP* scores on the commonly-used *IoU* range substantially differ from those on lower *IoU* ranges.

#### IoU versus point in mask

Considering the clinical goal of prioritizing the localization of polyps more than their boundaries, we compared the values of the aggregated metrics *Sensitivity, Positive Predictive Value (PPV), F1-Score, F2-Score* and *AP* using point-based localization criteria to the values obtained using *Box IoU*. *Point in Mask* returns a true positive (TP) if the center point of the predicted bounding box lies within the respective reference mask. The reference can be the segmentation mask, convex hull or bounding box. The result is shown in Table 3. *Point inside Reference* criteria yield higher scores across all metrics compared to *Box IoU* over most *IoU* thresholds. This especially holds true for detection *Sensitivity*. Note that the AP score for Center Point in Reference is 0.73 and thus comparable to the AP score for Box IoU criterion with a cutoff range of [0.05:0.5], namely 0.72.

**Table 3:** Point-based versus overlap-based localization criteria applied to the set of all six centers. Point-based criteria give rise to similar results while the *IoU* criterion consistently yields lower values. *IoU* intersection over union

Localization criterion	Box *IoU* = 0.5	Point inside box	Point inside mask	Point inside hull
*Sensitivity*	0.68	0.74	0.74	0.74
*PPV*	0.78	0.86	0.85	0.86
*F1-Score*	0.73	0.8	0.79	0.8
*F2-Score*	0.70	0.74	0.74	0.74
*AP*	0.65	0.73	0.73	0.73

### Alignment with clinical interest

In the presence of many sources of variability depending on the metric configuration, we conducted an experiment to determine which configuration aligns most with the clinical goal. We presented colonoscopy images of over 300 patients with their predicted bounding boxes to three gastroenterologists, one with over five years and two with over ten years of experience, who rated the predicted boxes as (clinically) “useful” or “not useful”. Each clinician was responsible for one third of the images and each image was only rated once.

**Fig. 7:**
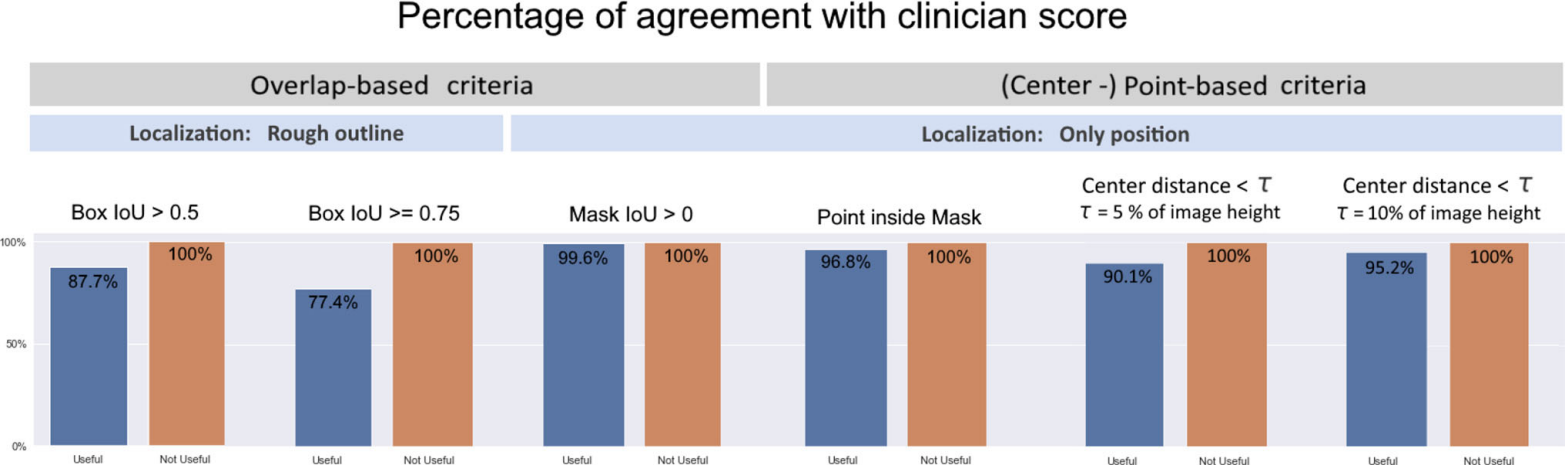
Agreement of common localization criteria with clinicians’ ratings. Predictions rated as “not useful” by clinicians were rejected by all criteria without exception. However, especially overlap-based localization criteria yielded a high proportion of false negatives that clinicians would have classified as “useful”. Almost perfect agreement was achieved by the metric *Mask IoU* > 0. Details on the analyzed localization criteria can be found in Appendix A

**Fig. 8:**
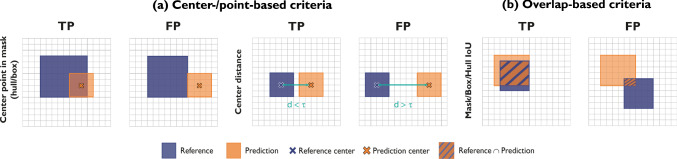
Localization criteria can be *point-based* or *overlap-based* depending on whether the user is mainly interested in the position or in the rough outline of an object. *Point in Mask* returns a TP if the center point of the predicted bounding box lies within the respective reference mask. The reference can be the segmentation mask, convex hull or bounding box. Center distance criterion determines a TP if the distance $d$ between prediction and reference centers is within a range $\tau$. For overlap-based criteria, the result is a TP if the overlap lies above a certain threshold. Depending on whether the *IoU* is computed for a reference mask or an approximating bounding box, we refer to it as *Mask* or *Box IoU*. *IoU* intersection over union, *TP* true positive, *FP* false positive

**Fig. 9:**
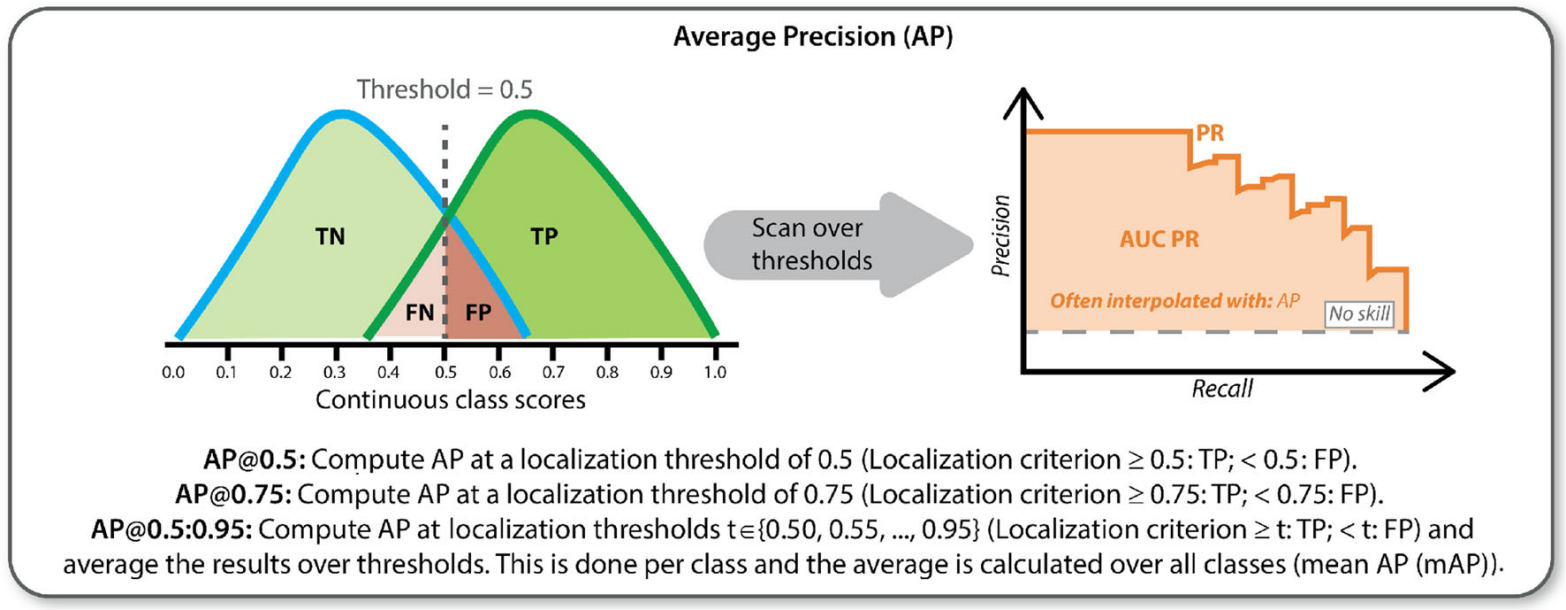
Pictorial representation of the *AP* metric. *AP* average precision

In order to assess the agreement of certain metric configurations with the clinician score, we plotted the number of predictions that met the criterion as a fraction of the predictions rated as “useful”, as well as the number of predictions not meeting the criterion as a fraction of predictions rated as “not useful”. We applied overlap-based and point-based criteria and highlighted the localization granularity that they focus on (rough outline or only position). The result can be seen as a bar plot in Fig. 7. All predictions clinically rated as “not useful” were rejected by all localization criteria. Criteria that focus only on position yielded a higher agreement with the “useful” score than those that localize based on overlap using rough outline. In other words, currently used localization criteria, which put a focus on object outlines through relatively strict IoU localization criteria may not be well-suited for reflecting the clinical interest (Figs. 8, 9 ).

### Generalizability of results

**Fig. 10:**
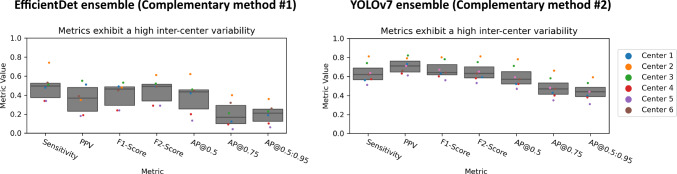
Complementary experiments: Sensitivity to center. Consistent with the result in Fig. 2, both complementary algorithms showcase high variability between centers for each metric. For example, the *AP@0.5:0.95* ranges from [0.06, 0.36] in the case of the EfficientDet ensemble, while the range is [0.31,0.59] for the YOLOv7 ensemble. *PPV* positive predictive value, *AP* average precision

**Fig. 11:**
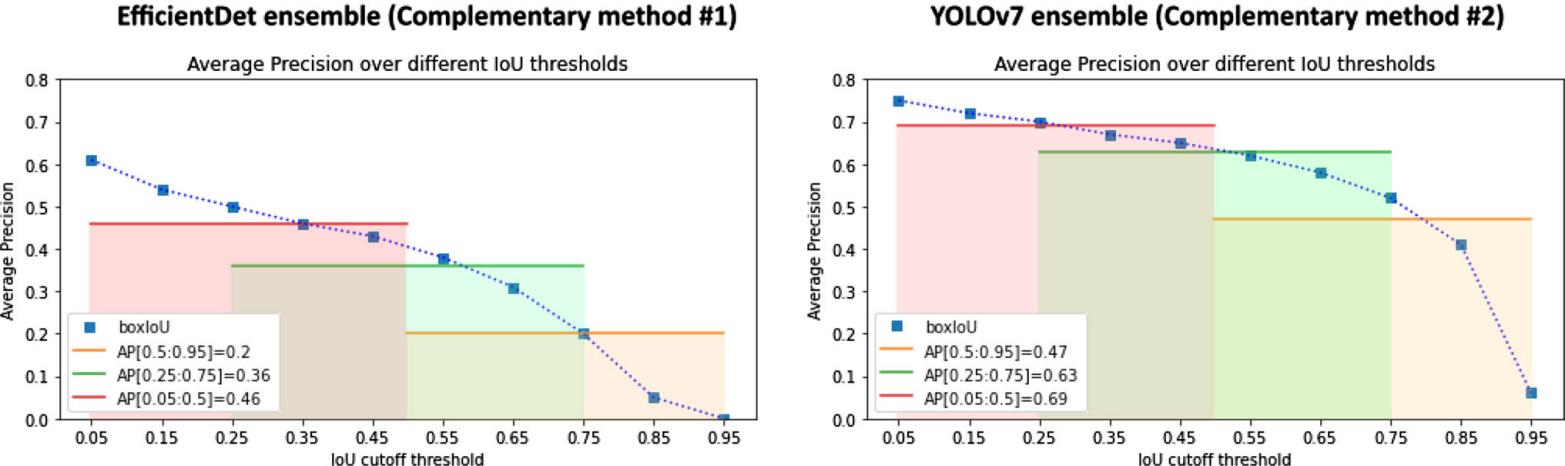
Complementary experiments: *AP* over different *IoU* thresholds. Coherent with the observation in Fig. 6, both complementary methods show that a lower *IoU* range results in a higher *AP* score. *AP* average precision, *IoU* intersection over union

As the present work is about performance variability rather than absolute performance we assumed that a high number of images (*n* = 1512), objects (*n* = 1386) and centers (*n* = 6) involved in the analyses is the key to ensure drawing solid conclusions. Specifically, we hypothesized that a state-of-the-art method applied to such a large number of cases would yield a representative distribution of output boxes. To confirm this hypothesis, we have repeated our analyses with two complementary algorithms based on different neural network backbones. (1) EndoCV 2021 winner: We used the open-source implementation (https://github.com/GorkemP/EndoCV2021-EfficientDet-Pytorch) of the EndoCV 2021 polyp detection challenge winner [19], which is based on an ensemble of EfficientDet networks and (2) YOLOv7 ensemble: To reflect the new state of the art in object detection (note that the EndoCV 2022 challenge took place in early 2022) we implemented an ensemble of YOLOv7 [20] models, which are currently considered the state of the art in object detection in terms of speed and accuracy. Both new methods were trained, optimized and tested on the same data splits as the EndoCV 2022 winner. As illustrated in Appendix B, Figs. 10, 11 and Tables 4, 5, in terms of the trend (relative values) the results are in very strong agreement with the results of the EndoCV 2022 winner (only absolute values differ).

## Discussion

To our knowledge, we were the first to systematically investigate the variability of polyp detection performance resulting from various validation design choices. The following key insights can be derived from our experiments:

1. *Performance results are highly sensitive to various design choices* Our experiments clearly demonstrate that various validation design choices have a substantial effect on the performance computed for object detection algorithms according to popular metrics. These range from the choice of test set to the specific metric configuration used. While the effect of using different classification metrics may be increasingly well-understood [15], we believe that common metrics, such as *AP*, are often regarded as black boxes and the effect of the various hyperparameters remains poorly understood. Our findings clearly suggest that hyperparameters—specifically the localization criterion and the corresponding threshold—should not indiscriminately be adopted from other work, but carefully be chosen to match the domain need.
2. *Common metric configurations do not reflect the clinical need* According to a usefulness assessment of polyp predictions from over 300 patients by three clinicians from different hospitals, commonly used localization criteria that are popular in the computer vision community do not reflect the clinical domain interest when deciding whether a prediction should be assigned a true positive or false positive. This holds specifically true for the international competitions that have been conducted in the context of Polyp detection. The community should therefore revisit the question of whether a good object detection method must necessarily yield a good outline of a polyp. Restricting the need to just localizing a polyp via its position (reflected by the requirement of $IoU > 0$, for example) might better approximate the clinical need and at the same time overcome problems resulting from suboptimal *IoU* thresholds.
3. *Common hyperparameters may be too restrictive* Our visual examples (Fig. 5) demonstrate that even fairly well-localized polyps feature an *IoU* below the commonly used threshold of 0.5, resulting in them being considered a miss even though a clinician might find the prediction useful. The community may therefore want to reconsider commonly used threshold ranges and use a broader range (see Fig. 6b).
4. *Comparison of performance across datasets can be largely misleading* Our work finds that detection performance depends crucially on the polyp sizes. Hence, even if the prevalences of polyps across centers are similar, comparison of algorithm results can be largely misleading in case of different polyp size distributions.

The closest work to ours was recently presented by Ismail et al. [21] outside the field of deep learning. They provide anecdotal evidence on the non-comparability of confusion matrices between different methods, but do not analyze common multi-threshold metrics such as *AP* or popular localization criteria that serve as the basis for popular classification metrics. Other related work focused on providing benchmarking data sets [2] or showing limitations of metrics for clinical use cases outside the field of polyp detection [7, 22, 23].

We purposefully investigated only the task of polyp detection (and no other object detection tasks) because performance variability is application-specific and metric hyperparameters should be adjusted to the clinical interest [15]. Future work could be directed to challenging current metric configurations also for other medical detection tasks.

A limitation of our study can be seen in the fact that we reported our findings only on a single data set [12]. However, this data set comprises images from six centers and can therefore be seen as sufficiently representative for the scope of our research question. Our results clearly show that validation results are not comparable unless a method has been tested on the same data with the exact same metric hyperparameters. Future work could investigate the stability of challenge rankings as a function of metric configurations. Finally, there are several other factors related to performance assessment that we did not prioritize in this work. These include the assignment strategy, the prevalence as well the confidence threshold in the case of counting metrics. Future work could hence explore the impact of these factors.

**Table 4:** *IoU* versus *Point in Mask* results of complementary algorithms

Localization criterion	Box *IoU* = 0.5	Point inside box	Point inside mask	Point inside hull
EfficientDet ensemble (Complementary method #1)				
*Sensitivity*	0.49	0.61	0.59	0.59
*PPV*	0.36	0.46	0.44	0.44
*F1-Score*	0.41	0.51	0.49	0.49
*F2-Score*	0.45	0.56	0.54	0.54
*AP*	0.37	0.49	0.47	0.47
YOLOv7 ensemble (Complementary method #2)				
*Sensitivity*	0.64	0.76	0.75	0.76
*PPV*	0.71	0.78	0.77	0.77
*F1-Score*	0.67	0.77	0.76	0.76
*F2-Score*	0.65	0.77	0.76	0.76
*AP*	0.59	0.74	0.73	0.73

Consistent with the observation shown in Table 3, point-based criteria give rise to similar metric values, while the Box *IoU* criterion consistently yields lower values. *IoU* intersection over union, *PPV* positive predictive value, *AP* average precision

**Table 5:** Sensitivity to polyp sizes of complementary algorithms

Metric	*AP@0.5*			*AP@0.5:0.95*		
Polyp size	Small	Medium	Large	Small	Medium	Large
*EfficientDet ensemble (Complementary method #1)*						
Center 1	0.12	0.2	0.5	0.07	0.08	0.22
Center 2	0.00	0.39	0.65	0.00	0.25	0.38
Center 3	0.02	0.24	0.59	0.01	0.12	0.29
Center 4	0.03	0.0	0.24	0.02	0.0	0.13
Center 5	0.08	0.08	0.17	0.03	0.04	0.08
Center 6	0.00	0.03	0.56	0.00	0.23	0.32
All centers (SD)	0.04 (0.05)	0.2 (0.14)	0.45 (0.2)	00.2 (0.03)	0.12 (0.1)	0.24 (0.12)
*YOLOv7 ensemble (Complementary method #2)*						
Center 1	0.12	0.41	0.59	0.07	0.29	0.45
Center 2	0.00	0.53	0.81	0.00	0.33	0.63
Center 3	0.12	0.57	0.83	0.06	0.36	0.64
Center 4	0.07	0.49	0.58	0.01	0.26	0.44
Center 5	0.22	0.41	0.58	0.13	0.27	0.39
Center 6	0.00	0.46	0.69	0.00	0.3	0.55
All centers (SD)	0.09 (0.08)	0.48 (0.06)	0.68 (0.12)	0.05 (0.05)	0.3 (0.04)	0.52 (0.11)

Consistent with the observation shown in Table 2, the results suggest that there are strong effects of polyp size on the AP values. For both methods and across all centers, smaller polyps have a lower *AP@0.5* and* AP@0.5:0.95* score than medium sized polyps, while larger polyps generally score higher. *AP* average precision, *SD* standard deviation

In conclusion, our study is the first to systematically demonstrate the sensitivity of commonly used performance metrics in deep learning-based colon cancer screening to a range of validation design choices. In showing clear evidence for the disparity between commonly used metric configurations and clinical needs, we hope to raise awareness for the importance of adapting validation in machine learning to clinical relevance in general, and spark the careful reconsideration of common validation strategies in automatic cancer screening applications in particular.

## Data Availability

Not applicable.

## A Appendix

In this work, we distinguish *point-based* and *overlap-based* localization criteria (see Fig. 8). Point-based criteria include the *(Center) Point in Mask* criterion, which defines a TP once the center point of the predicted object or bounding box lies inside of the reference mask (alternatively, reference box or reference hull) and the *center distance*, which calculates the distance between the reference and predicted center points. This criterion defines a TP if the distance is below a user-defined threshold. Overlap-based criteria compute the overlap between the reference and the prediction, in our case done by the *Mask* or *Box IoU*. This criterion returns a TP if the *Mask/Box IoU* is greater than a user-defined threshold. A specific case is the *Mask IoU* > 0 criterion, which returns a TP once only a small amount of pixels (e.g., a single pixel) of the prediction overlaps with the reference.

We further utilize the *AP* as a validation metric of the detection performance. In practice, *AP* is typically calculated at a specific *IoU* threshold (e.g., *AP@0.5* for a threshold of 0.5) or a range of thresholds. The metric is calculated by scanning over different decision thresholds and calculating the Sensitivity (Recall) and Precision (PPV) at the respective thresholds. The points are connected to form a diagram, as depicted in Fig. 9.

## B Complementary experiments

This section presents the results corresponding to our generalizability experiment described in Sect. 3.4
